# Risk-sensitive foraging in a tropical lizard

**DOI:** 10.1098/rsbl.2024.0628

**Published:** 2025-02-19

**Authors:** Avik Banerjee, Maria Thaker

**Affiliations:** ^1^ Centre for Ecological Sciences, Indian Institute of Science, Bengaluru, India

**Keywords:** reptile, starvation, energy budget, risk averse, risk prone, optimal foraging theory

## Abstract

Foraging opportunities can be unpredictable. When foragers face a choice between resources that vary in predictability, foraging decisions not only depend on the profitability of food but also on their physiological state. This risk-sensitive foraging approach, in which animals take greater foraging risks when starving, remains relatively untested in reptiles compared with other taxa. We tested the risk-sensitive foraging theory in the tropical lizard, *Psammophilus dorsalis*, by manipulating energy budgets (satiated versus 48 h starved) and measuring foraging preferences for options that differed in rewards: constant (two mealworms) versus variable (zero or four mealworms). We found that satiated lizards were risk averse to variability in reward amounts and chose the constant food option more frequently than the variable option. In contrast, starved lizards were risk-prone and chose the variable reward option more often than the constant one. At the end of foraging trials, these strategies resulted in both starved and satiated groups achieving similar net resource gains. As new support for risk-sensitive foraging in a tropical reptile species, these results provide insight into how resource uncertainty influences foraging strategies. For lizards in the tropics, which have high-energy requirements year-round, risk-sensitive foraging could be an effective strategy in stochastic environments.

## 1. Introduction

Risk-sensitive foraging theory posits that foraging choices not only depend on the reward probability of food options but also on the variability and predictability of those options [1,2]. This is unlike models of optimal foraging, which are indifferent to uncertainty risk and assumes an all-or-nothing approach to foraging choices, where animals forage in a way that maximizes a fitness currency, which is typically the net rate of energy gain from an expected food source [3–5]. In the wild, however, food resources are variable and unpredictable and thus, the profitability of food rewards is not always certain [1]. Animals should therefore adjust their foraging decisions based not only on the profitability of food but also other complex factors, including their current physiological state and the predictability of food resources in the natural environment [3,6,7].

Risk-sensitive foraging approach takes into account the risk associated with variability in food reward amounts [1]. Caraco *et al.* [2] provided the first empirical evidence for risk-sensitive foraging in birds by experimentally manipulating energy budgets and foraging options of yellow-eyed juncos. They found that when faced with foraging choices that were predictable (i.e. had constant rewards) or unpredictable (i.e. had variable rewards), birds were either risk averse or risk prone depending on their energy budgets [2]. The energy budget rule provided a functional explanation for risk-sensitive foraging behaviour [8,9]. For example, in the experiment by Caraco *et al.* [2] when birds were maintained at a positive energy budget, wherein their energy reserves were higher than required for survival, they were risk averse, preferring to choose the constant (but low reward) food option over the variable (but high reward) one. In contrast, when birds were at a negative energy budget, wherein their energy reserves were insufficient to ensure survival, they were risk prone, switching their foraging preference to the variable food option which had the potential to provide greater rewards [10,11]. Since Caraco *et al.* [2], numerous studies have tested the risk-sensitive foraging theory in insects [12], fishes [13], birds [14] and mammals [15]. However, not all studies find consistent support. Several studies have found the opposite pattern wherein animals show risk-averse responses even when they are starving or show no sensitivity to uncertainty risk of foraging options [11,16,17].

Foraging behaviour of reptiles has been studied for many decades, and it is clear that reptiles make optimal diet choices based on prey type [18], foraging strategy [19] and predation pressure [20]. Whether reptiles make risk-sensitive foraging decisions based on the predictability and variability of food resources remains unknown. Because reptiles can have remarkably low-field metabolic rates [21], high lipid storage capacity [22] and multiple physiological adaptations for torpor [23], they can combat energetic shortages or starvation risk in the wild. These adaptive traits that are beneficial in resource-scarce conditions like winter may not be optimal for reptiles that live in the tropics. In the tropics, carnivorous reptiles have rapid growth rates and high reproductive frequency [24–26] which are energy-intensive. They also experience relatively high-temperature conditions year-round and thus have continuous thermal acclimation responses that are also energetically costly [27–29]. Thus, the sustained energetic requirements that reptiles in the tropics (e.g. [30]) have all year-round should result in the selection for the ability to assess uncertainty and modulate foraging choices to meet daily energy demands.

In this study, we tested the risk-sensitive foraging theory in a tropical agamid lizard, *Psammophilus dorsalis*, which is a large-bodied, sexually dimorphic species found in semi-arid landscapes of peninsular India. We manipulated the starvation (or energetic) state of individuals (i.e. satiated versus starved), and measured the foraging choices of these individuals when they were provided with a predictable but low-profitable food source (i.e. offering constant reward amounts) versus an unpredictable but potentially high-profitable food source (i.e. offering variable reward amounts). Based on the energy budget rule, we predicted that satiated lizards, that are at a higher energetic state (or positive energy budget), will prefer the constant food source (i.e. safe choice), whereas starved lizards, that are at a lower energetic state (or negative energy budget), will prefer the variable food source (i.e. risky choice). This test of risk-sensitive foraging in a reptile not only adds a novel taxon to the field but also allows us to better understand how tropical ectothermic vertebrates could be optimizing foraging preferences when faced with variability in food rewards.

## 2. Methods

### (a) Capture and housing

Adult lizards of both sexes (*n* = 23 males and 27 females) were captured from rocky habitats in Karnataka, India, by lassoing and housed individually in glass terraria (60 × 30 × 25 cm) in the laboratory. Terraria were placed in a dedicated lizard room maintained at ambient temperature (approx. 28°C) with a 12 h light/dark cycle. Terraria was also fitted with a 100 W basking bulb approximately 30 cm overhead that was turned on for 2 h each day. Wild-caught lizards were acclimated to laboratory conditions for 4−5 days, during which they were provided with *ad libitum* water and four mealworms per day. Lizards that did not feed on mealworms during the acclimation period (two males and one female) were excluded from subsequent behavioural trials. All trials were conducted in the housing terraria of lizards and were video recorded via cameras mounted above each terrarium. Body mass (g) measured on a digital weighing scale and snout-to-vent length (SVL) measured with a ruler (mm) were recorded for all lizards before and after the experiment. At the end of experiments, lizards were marked to prevent recapture with non-toxic ink on their ventral surface and released at their site of capture. All experiments were conducted during the peak activity months of the lizard between March to September in 2019 and 2020.

### (b) Behavioural experiment

Lizards were required to make foraging choices based on their energetic state and the risk associated with choosing between two foraging options that provided different reward amounts. To manipulate the energetic state of lizards, we randomly assigned individuals of both sexes to a ‘satiated’ group (*n* = 13 females (mean ± s.e. = 90 ± 2.7 mm SVL) and 11 males (124 ± 2 mm)) or a ‘starved’ group (*n* = 12 females (93 ± 2.9 mm) and 10 males (120 ± 3.8 mm)), after the training phase (detailed below). Lizards in the ‘satiated’ group were provided with foraging choices every day and those in the ‘starved’ group were provided with foraging choices every third day with a 48 h starvation period in-between. Given similar energy expenditures between the groups but different resource intake opportunities, the energetic state of lizards in the starved group was expected to be lower than those in the satiated group.

There were two phases of the experiment, a training phase followed by a testing phase. Lizards were first given an associative training regimen which allowed them to familiarize themselves to possible reward outcomes of the two foraging options. We used associative training trials in which we coupled colours of Petri dishes (i.e. blue or green) with specific foraging rewards. The dish that was designated as ‘constant’ had two mealworms as the foraging reward in every trial and the dish designated as ‘variable’ had either zero or four mealworms at equal probabilities (*p =* 0.5). Therefore, for any given trial (or ‘feeding opportunity’), the ‘variable’ food source had potentially higher net profitability (four mealworms) than the ‘constant’ food source (two mealworms). During both the training phase and the testing phase, two trials were conducted each day at 08.30–11.30 h and 13.30–17.00 h, with an interval of a minimum 2 h between trials.

### (c) Training phase

**Figure 1.**
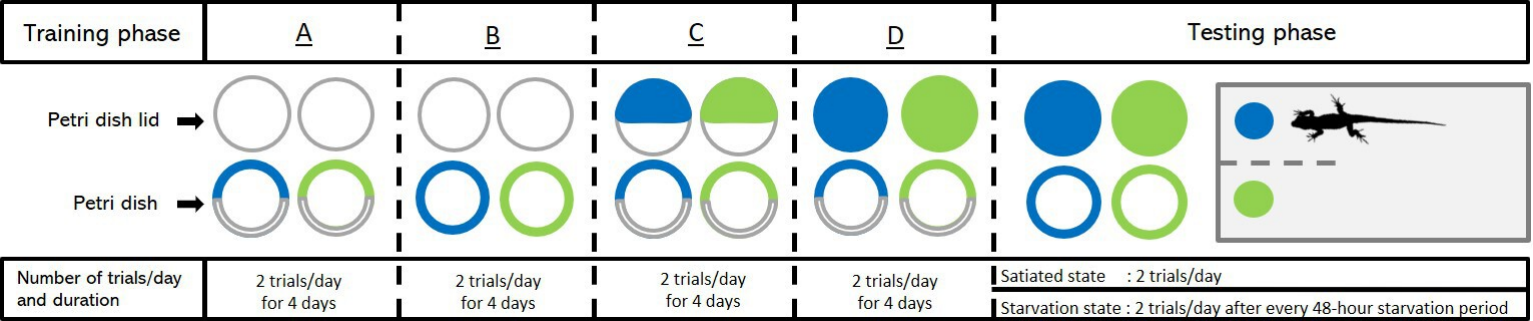
The training paradigm consisted of four phases (A–D), followed by a testing phase. During the training phase (A–D), the opaque covering of Petri plates was gradually increased until fully covered and lizards were given access to mealworms at the end of trial regardless of their choice. Black dashed lines mark the transition from one phase to the next, which happened once the lizard successfully approached the Petri plates and attempted to remove the lid in at least four trials. During the testing phase, lizards were either in the satiated group (two trials per day for 28 total trials) or the starved group (two trials after every 48 h starvation period for 28 total trials). The grey rectangular schematic shown in the testing phase depicts a lizard making a choice and the grey dashed line within it depicts a physical barrier between the two Petri plates.

The purpose of the training phase was to have lizards associate a particular colour with a corresponding food reward (constant or variable). In each trial, lizards were provided with two Petri dishes that were partially covered with a designated opaque colour. The coverage of opaque colour over the Petri dish and lid was gradually increased over subsequent training phases (A–D, see figure 1), such that visibility of mealworms through the dishes would decrease over time. This protocol ensured gradual elimination of prey visual cues as a motivator for lizards to approach the Petri dishes, and the subsequent association of colour on the Petri dish with their reward. This was essential as lizards would be expected to make foraging choices in the testing phase based on their energetic state and expected food reward in each dish and not prey visual cues. We randomized the assignment of colour (blue or green) to type of food reward (constant or variable) between lizards and randomized the position of the dishes within the terrarium in each trial. Each trial lasted for 30 min, and we ran a total of eight trials in each of the training phases A–D. In all trials, Petri dishes were covered so that lizards were able to see the food rewards but were not able to access them until the end of the trial. A trial was considered successful if the lizard approached a Petri dish and attempted to feed on the mealworms. Lizards that successfully completed at least four trials of a phase were moved to the next phase in the training. Lizards that failed to satisfy the above criteria (*n* = 1 female) after eight training trials in a phase were excluded from the experiment.

At the end of each trial, we randomly opened the lid of one of the Petri dishes and removed the other Petri dish from the terraria so that lizards were allowed to access the food rewards. Both the ‘constant’ and ‘variable’ feeding dishes were opened in equal probability at the end of the trials during the training phase. We ensured that lizards were made to access all possible rewards, i.e. zero, two or four mealworms, during each training phase, such that rewards gained over time across both the feeding options were equal. This controlled for any possible choice bias for a reward type during the training phase. These training trials were considered ‘no-choice’ trials since the opening and access to rewards from either constant or variable food sources were independent of the lizard’s foraging choice.

### (d) Testing phase

Lizards that successfully completed all the training phases proceeded to the testing phase. During this phase, lizards were maintained at either a satiated or starved state. Lizards in the satiated group were provided with two foraging choice trials every day, and those in the starved group were provided with two foraging choice trials every 3 days, with a 48 h starvation period in-between trial days. This protocol was followed until a total of 28 choice trials were conducted for both the groups. During the starvation period of 48 h, only *ad libitum* water was available to lizards, but no food was provided.

During the testing phase, the Petri dishes were completely covered by the designated colour so that lizards were unable to see food rewards. Foraging choices in this phase were expected to be based only on energetic states and the associative training. A physical barrier (20 × 10 cm white foam board) was placed in-between the two feeding dishes to help ascertain the lizard’s choice (see figure 1). The position of the feeding dishes (constant and variable) on either side of the barrier was randomized across trials. The food reward for the ‘variable’ feeding dish (i.e. either zero or four mealworms) was also randomly selected for each trial at equal probability (*p* = 0.5), such that the mean rewards from both constant and variable feeding dishes were equal at the end of experiment. We recorded the first foraging choice made by the lizard (i.e. constant or variable) within 30 min of Petri dish presentation. If no foraging choice was made during the 30 min trial time, we recorded the trial as ‘no choice’ and proceeded to the next trial. Lizards were only allowed to access the mealworms from their chosen feeding dishes during the testing phase.

### (e) Statistical analyses

We used a generalized linear mixed-effects model (GLMM with a Poisson distribution for count data) to test for differences in the number of foraging choices made by lizards in the satiated and starved groups during the testing phase (using the R package *lme4,* [31]). In the models, the number of choices made was the response variable and the choice type (i.e. constant and variable), lizards’ energy state (i.e. satiated or starved) and sex (i.e. female or male) were predictor variables, with a three-way interaction. We also added animal ID as a random effect to account for repeated measures. *Post hoc* pairwise comparisons were done when relevant (using *emmeans* [32]), with Tukey’s HSD corrections. We tested the effect of energy state and sex with an interaction effect on the number of mealworms eaten during the testing phase, using a GLM with a Poisson distribution, and on the change of body mass (end mass – start mass) using a GLM with a Gaussian distribution. Additionally, we used a variance test (*F*-test) to determine the difference in variability of body mass changes between satiated and starved groups for males and females separately. All statistical analyses were done using R, v. 4.4.0. (available from the Dryad Digital Repository [33], Supplement 1: For raw data, Supplement 2: For R codes to run our analyses and Supplement 3: Video of a lizard in a testing trial).

## 3. Results

**Figure 2.**
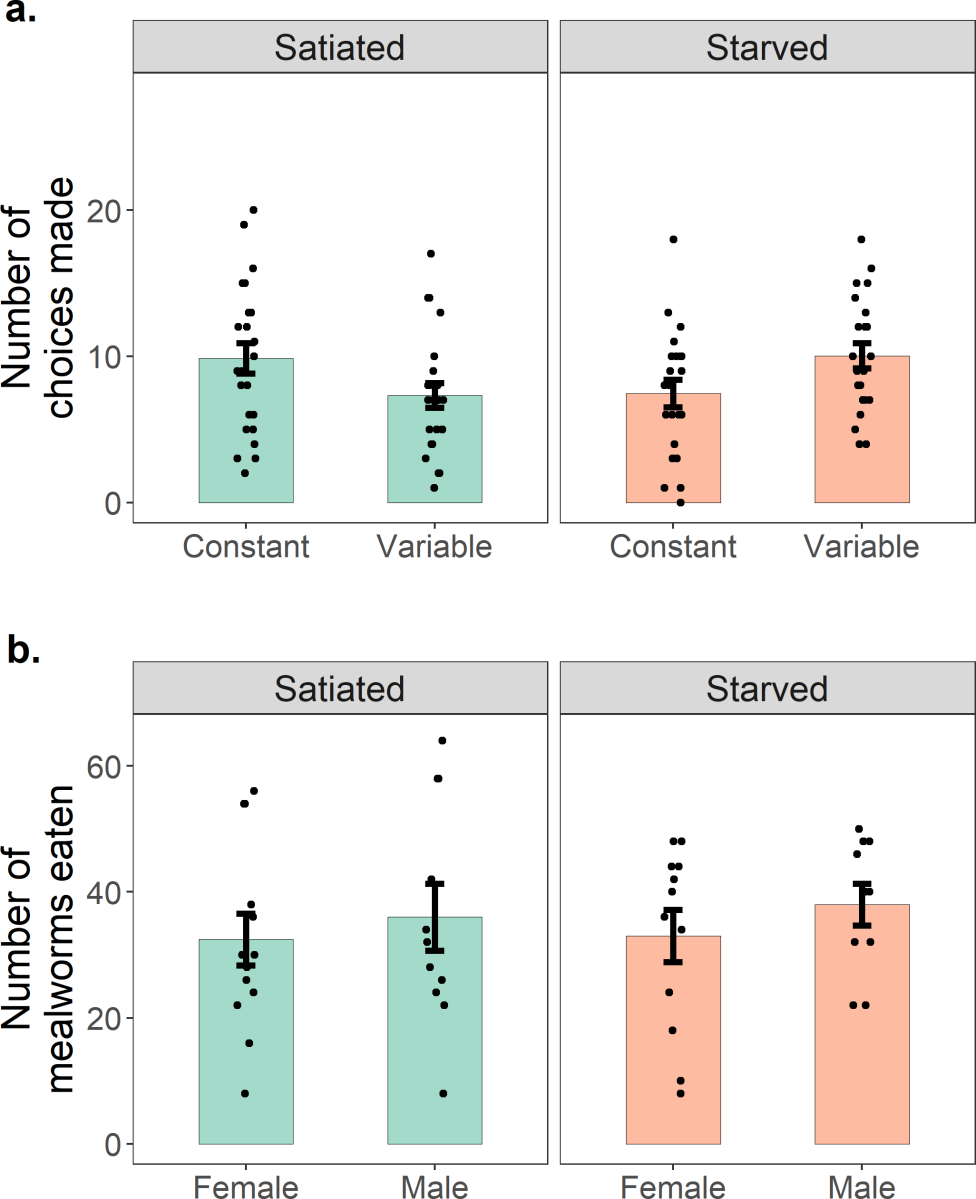
(a) Number of constant and variable choices made and (b) number of mealworms eaten, by males and females of *Psammophilus dorsalis* from the satiated (green) and 48 h starved (orange) groups. Bar plots with error bars represent mean ± s.e.

We found that the energy state (satiated or starved) of lizards significantly affected the number of constant and variable choices made regardless of the sex of the individual. Lizards in the satiated state chose the (safe) constant food option (mean ± s.e. = 9.9 ± 1 choices) significantly more often (Z = 2.985, *p* < 0.01, figure 2a) than the (risky) variable food option (7.3 ± 0.8 choices). In contrast, lizards in the starved group choose the variable food option (10 ± 0.9 choices) significantly more often (*Z* = −2.948, *p* < 0.01, figure 2a) than the constant food option (7.5 ± 0.9 choices). The sex of the individual did not have a significant effect on the number of choices made, i.e. males and females made a similar number of constant or variable food choices in both the satiated (constant choices: *Z* = −0.216, *p* = 0.8 and variable choices: *Z* = −1.228, *p* = 0.2) and 48 h starved (constant choices: *Z* = 0.001, *p* = 0.9 and variable choices: *Z* = −0.754, *p* = 0.5) groups. There was a negligible effect of ‘animal ID’ on number of choices made for both satiated and starved groups (GLMM random effect: s.d. < 0.5).

**Figure 3.**
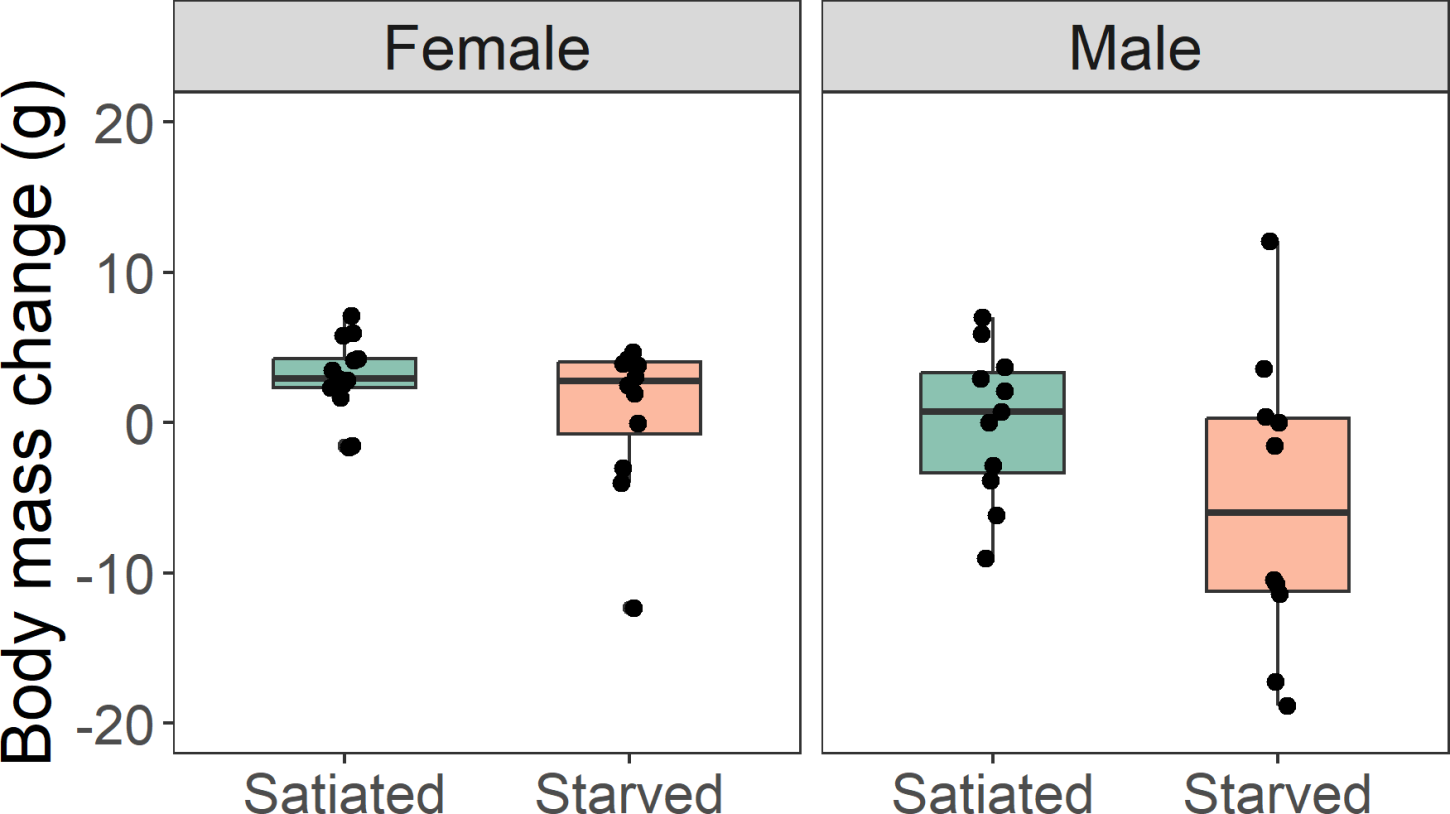
Change in body mass of both sexes of *Psammophilus dorsalis* from the satiated (green) and 48 h starved (orange) treatment groups. Change in body mass (g) was calculated as the difference between final (after experiment) and initial (before experiment) mass of lizards.

Even though satiated and 48 h starved-state lizards varied in the number of constant and variable foraging choices made during the testing phase, lizards of both sexes (*Z* = 1.479, *p* = 0.1) and from both energy state groups (*Z* = 0.235, *p* = 0.8) gained similar number of mealworm rewards (mean ± s.e., satiated state females = 32 ± 4.1 and males = 36 ± 5.3 mealworms; and starved state females = 33 ± 4.2 and males = 38 ± 3.3 mealworms) during the testing phase (figure 2b). There was no interaction effect between energy state and sex on the number of mealworms eaten (*Z* = 0.375, *p* = 0.7). When we consider body mass change during the experiment (mass at the end of experiment − mass at the start of experiment), we find that starvation state and sex of the lizards did not significantly affect body mass change, either individually (state: *t* = −0.97, *p* = 0.3; sex: *t* = −1.24, *p* = 0.2) or as an interaction effect (*t* = −0.895, *p* = 0.4). Thus, lizards of both sexes and from both the satiated and starved groups showed similar body mass changes. It is worth noting here that although body mass changes did not differ between energy state groups and sex of lizards, the variance in mass change of the starved group lizards was greater than that of the satiated group lizards for both females (*F*_(12,11)_ = 0.27, *p* = 0.03) and males (*F*_(10,9)_ = 0.26, *p* = 0.05). Overall, lizards in the starved group showed greater variation in body mass changes than the satiated group (figure 3).

## 5. Discussion

Animals can optimize their foraging decisions based not only on available time, nutrient requirements, competition and predation risk, but also depending on variability in reward amounts in their natural environment [1,6,34]. To the best of our knowledge, our experiment provides the first evidence for risk-sensitive foraging in a reptile from the tropics. We find that the lizard, *P. dorsalis*, is risk-sensitive to the variability in reward amounts when choosing between two foraging options that provided variable rewards. In the current experiment, we manipulated the starvation (or energetic) state of lizards and found that satiated lizards, that were allowed to make foraging choices daily, preferred the predictable or constant food option. In contrast, when lizards were only allowed to make foraging choices every third day after undergoing 48 h periods of starvation, they chose the unpredictable or variable food option more frequently. In other words, satiated lizards were risk-averse and starved lizards were risk-prone to variability in reward amounts.

These results are in accordance with past research on risk-sensitive foraging that also manipulates starvation levels of animals in order to alter their energetic states [2,13,35]. Although we did not explicitly measure the energy budget of lizards, our study design ensured that energetic losses were incurred during starvation periods, and thus, lizards in the starved group were expected to be at a lower energetic state than those in the satiated group [36]. Our results find support for the predictions of the energy budget rule. When lizards are at a higher (positive) energetic state, being risk-averse seems optimal as it decreases the probability of starvation by avoiding the variability in the rate of returns. Whereas, when lizards are starved, selecting for variability in the rate of returns increases the chances to avoid starvation and therefore, being risk-prone seems to be the optimal behaviour [1,7–9].

Risk-sensitive foraging behaviour is likely to vary depending on the species, context and type of reward [1,37–39]. Some studies, especially in rats, have shown sex-specific differences in risk sensitivity, which was attributed mainly to differences in biological requirements between sexes [40,41]. In our study, male and female lizards from both the starved and satiated groups were not different in their risk-taking behaviour, indicating that during the active season, both sexes were similarly limited in foraging gains or incurred similar starvation costs. Notably, females in our study were not gravid; but if they were developing eggs, the higher energetic demands might have resulted in sex-specific differences in risk sensitivity. We also found that starved lizards made more risky choices but did not gain higher net reward amounts, i.e. total number of mealworms eaten (figure 2b). This result was not surprising as net profitability from both the foraging options was kept equal in the study (i.e. the number of mealworms in the constant and variable options over all the trials of the testing phase (*n* = 28) was the same). Although the changes in body mass were not statistically different between satiated and starved lizards at the end of the experiment, lizards in the starved group showed higher variance in body mass change than those in the satiated group (figure 3). Inter-individual variation in physiological response to starvation may explain this variance [36,42], but it is interesting to note that mass change was most variable for males in the starved group. Thus, for males, risk-prone foraging decisions are unlikely to overcome the energetic costs incurred by being the larger-bodied sex.

Risk-sensitive foraging studies on ectotherms have been scarce, limited to insects and fishes [12,13]. Some of this taxonomic bias comes from the assumption that energetic demands of endotherms exceed those of similar-sized ectotherms, and therefore, endotherms are expected to be more sensitive to foraging risk. However, the hot and aseasonal environment that some tropical ectotherms experience requires sustained metabolic processes that are energetically intensive [27–29]. Tropical ectotherms, including *P. dorsalis*, are also under increasing threat from climate warming and habitat loss [43,44], and when environments become more resource scarce or more unpredictable, species like this could face foraging limitations as well as warming-induced activity restrictions [45,46]. Being risk-sensitive can be an effective and potentially optimal foraging strategy for *P. dorsalis* and other tropical ectotherms to avoid starvation costs and maintain homeostasis under such conditions [3,39,47]. In summary, variability in reward amounts resulted in variability in choice and risk-sensitive foraging behaviour in the tropical lizard, *P. dorsalis*. Our study provides novel evidence towards understanding optimal foraging strategies in reptiles faced with resource uncertainty in a stochastic environment. It also calls for more studies on ectotherms from tropical environments to better understand how physiological variation can influence energy budgets and therefore the ability to assess and consider uncertainty risk in foraging.

## Data Availability

All data presented in this manuscript and the code used to analyze the data are available from the Dryad Digital Repository [33].
