# Correction: ‘Risk-sensitive foraging in a tropical lizard’ (2024), by Banerjee and Thaker

**DOI:** 10.1098/rsbl.2025.0106

**Published:** 2025-04-09

**Authors:** Avik Banerjee, Maria Thaker

**Affiliations:** ^1^ Centre for Ecological Sciences, Indian Institute of Science, Bangalore, Karnataka 560012, India

*Biol. Lett.* **21**: 220240628 (Published online 19 February 2025). (https://doi.org/10.1098/rsbl.2024.0628)

We found a typographical error in our published manuscript [1]. In the ‘Results’ section, we state: ‘In contrast, lizards in the starved group choose the variable food option (7.5 ± 0.9 choices) significantly more often (Z = −2.948, *p* < 0.01, figure 2a) than the constant food option (10 ± 0.9 choices)’. The values in the brackets are interchanged. The correct statement should read, ‘In contrast, lizards in the starved group choose the variable food option (10 ± 0.9 choices) significantly more often (Z = −2.948, *p* < 0.01, figure 2a) than the constant food option (7.5 ± 0.9 choices)’.

This typographical error does not affect any of our results or the discussion presented in the manuscript, and has been corrected on the publisher's website.
